# Clinical characteristics and prognostic impact of HER2-ultralow breast cancer and tumor-infiltrating lymphocytes (TILs)

**DOI:** 10.1186/s12885-025-15255-w

**Published:** 2025-11-28

**Authors:** Koji Takada, Shinichiro Kashiwagi, Mariko Nishikawa, Asuka Kochi, Chika Watanabe, Haruhito Kinoshita, Kana Ogisawa, Masatsune Shibutani, Tamami Morisaki

**Affiliations:** 1 Department of Breast Surgical Oncology, Graduate School of Medicine, Osaka Metropolitan University, 1-4-3 Asahi-machi, Abeno-ku, Osaka, 545-8585 Japan; 2 Department of Gastrointestinal Surgery, Graduate School of Medicine, Osaka Metropolitan University, 1-4-3 Asahi-machi, Abeno-ku, Osaka, 545-8585 Japan

**Keywords:** Breast cancer, HER2-low, HER2-ultaralow, Tumor-infiltrating lymphocytes, Neoadjuvant chemotherapy

## Abstract

**Purpose:**

HER2 expression is crucial in breast cancer classification and treatment. Traditionally, tumors were categorized as HER2-positive or HER2-negative, but HER2-low (IHC 1 + or 2 + without ISH amplification) has emerged as a new classification. Among HER2-negative cases, HER2-ultralow (≤ 10% faint HER2 staining) and HER2-null (completely HER2-negative) have been proposed. While differences between HER2-low and HER2-zero tumors are studied, *little is known about the clinical and prognostic characteristics of HER2-ultralow breast cancer*. This study *aimed to clarify* the clinical characteristics, immune microenvironment, treatment response, and prognosis of HER2-ultralow tumors, *with HER2-null and HER2-low tumors analyzed as comparators*.

**Methods:**

A retrospective analysis of 244 HER2-negative breast cancer patients treated with neoadjuvant chemotherapy (NAC) at Osaka Metropolitan University Hospital (2007–2018) classified tumors into HER2-low (41.0%), HER2-ultralow (36.1%), and HER2-null (23.0%). *Clinicopathological features*,* tumor-infiltrating lymphocyte (TIL) counts*,* pathological complete response (pCR)*,* and prognostic outcomes (disease-free survival [DFS] and overall survival [OS]) were evaluated*.

**Results:**

HER2-ultralow tumors showed significantly higher estrogen receptor (ER) positivity compared with HER2-null tumors (51.8% vs. 19.6%, p < 0.001), and also tended to have higher progesterone receptor positivity (p = 0.048). In contrast, HER2-null tumors were associated with younger age (median 50.0 vs. 56.0 years, p = 0.004) and higher TIL density (50.0% vs. 36.8%, p = 0.016). The overall pCR rate was 27.9%. DFS showed no significant differences among the three groups (p = 0.087), but OS was significantly worse in HER2-null compared with Not HER2-null tumors (p = 0.026, HR = 0.454). HER2-ultralow cases demonstrated an intermediate prognosis between HER2-low and HER2-null (OS comparison with HER2-null,>p= 0.101).

**Conclusion:**

HER2-ultralow tumors *represent a distinct subgroup characterized by higher hormone receptor positivity*, whereas HER2-null tumors were associated with younger age, higher TIL density, and poorer survival. *These findings emphasize the clinical significance of refining HER2-negative subclassification to distinguish HER2-ultralow*,* while acknowledging limitations of sample size and retrospective design*.

**Supplementary Information:**

The online version contains supplementary material available at 10.1186/s12885-025-15255-w.

## Background

In the therapeutic management of breast cancer, it is important to confirm the presence or absence of hormone receptor expression and human epidermal growth factor receptor 2 (HER2) receptor expression. HER2 status has traditionally been classified as either positive or negative by immunohistochemistry (IHC) and in situ hybridization (ISH) test. Although anti-HER2 targeted therapies such as trastuzumab and pertuzumab have demonstrated high efficacy in combination for HER2-positive breast cancer, no effect has been observed for negative cases [1, 2].

Recently, with the advent of novel antibody-drug conjugates (ADCs), a new concept of HER2 expression has been proposed in previously HER2-negative breast cancer. With the emergence of new ADCs, HER2-low breast cancer, *defined as IHC 1 + or IHC 2 + without amplification on ISH*,* has been recognized as a clinically relevant subgroup and showed a higher therapeutic effect than HER2-zero*,* which is completely negative (IHC 0)* [3–5].

Furthermore, among HER2-zero patients, more detailed subclassification has been proposed. The results of trastuzumab deruxtecan trials suggested differences in therapeutic effect between HER2-ultralow, *defined as tumors with ≤ 10% of tumor cells showing faint/weak membrane staining*, and HER2-null, *defined as tumors completely lacking HER2 staining* [6].

Although there have been recent reports describing differences in prognosis, clinical characteristics, and biology between HER2-low and HER2-zero breast cancers, there are still few reports specifically evaluating HER2-ultralow and HER2-null. Therefore, in this study, we evaluated the clinical characteristics and tumor immune microenvironment according to this refined HER2 classification in HER2-negative breast cancer patients treated with neoadjuvant chemotherapy (NAC), and retrospectively analyzed their therapeutic effect and prognosis.

## Methods

### Patients

The study included 244 HER2-negative breast cancer patients who underwent surgical treatment after NAC at the Osaka Metropolitan University Hospital between May 2007 and December 2018. All patients had been pathologically diagnosed with breast cancer by core needle biopsy (CNB) or vacuum-assisted biopsy (VAB), and the specimens were confirmed to be HER2 negative by IHC. Specifically, HER2 expression was assessed by IHC using HercepTest II (Dako), and *cases with IHC 2 + were further confirmed as negative by ISH*.

In addition to HER2 expression, we evaluated estrogen receptor (ER), progesterone receptor (PgR), and Ki67 *index (with ≥ 20% defined as high)* by IHC. After the diagnosis of breast cancer, disease staging was performed by computed tomography (CT), ultrasonography (US), and bone scintigraphy. NAC was indicated according to tumor subtype and stage. The first half of NAC consisted of four courses of FEC100 (500 mg/m² fluorouracil, 100 mg/m² epirubicin, and 500 mg/m² cyclophosphamide) every 3 weeks. In the second half, 12 weekly courses of 80 mg/m² paclitaxel were administered [7–9].

After NAC, treatment response was assessed according to the Response Evaluation Criteria in Solid Tumors (RECIST) [10]. Surgery consisted of either mastectomy or breast-conserving surgery [11]. Pathological complete response (pCR) was defined according to the NSABP B-18 protocol as “the complete disappearance of the invasive components of the lesion with or without intraductal components, including in the lymph nodes” [12]. Standard adjuvant therapy was administered according to subtype and surgical procedure.

#### Pathological evaluation

HER2 expression and *tumor-infiltrating lymphocyte (TIL) counts* were evaluated using biopsy tissue at diagnosis. HER2 status was classified into three categories according to previous reports [13, 14]:

- HER2-low: IHC 1+ or IHC 2+ without amplification on ISH
- HER2-ultralow: ≤10% of invasive tumor cells showing incomplete and faint/weak membrane staining
- HER2-null: complete absence of membrane staining in invasive tumor cells Thus, HER2-ultralow was explicitly analyzed as a distinct subgroup, with HER2-null used as a comparator.

H&E-stained specimens were used for TIL evaluation, and TILs were defined as lymphocytes infiltrating the tumor stroma according to the International TILs Working Group 2014 [15]. TIL density was scored according to previous reports, with a cut-off value of 10% [16–19]. All pathological evaluations were performed by one or more pathologists with expertise in breast cancer. The median follow-up time was 2754 days (range, 149–5790 days) from surgery.

### Statistical analysis

All statistical analyses were performed using SPSS version 28.0 (IBM Inc.). For evaluating correlations between clinicopathological features, *Pearson’s chi-square test or Fisher’s exact test*,* as appropriate*,* were applied*. Survival curves for DFS and OS were estimated using the Kaplan–Meier method and compared using the log-rank test. Hazard ratios (HRs) and 95% confidence intervals (CIs) were calculated using the Cox proportional hazards model, and multivariate analysis was performed using a Cox regression model. A p-value < 0.05 was considered statistically significant.

### Ethics statement

This study was conducted at Osaka Metropolitan University in Japan, following the principles of the Declaration of Helsinki. The study protocol was approved by the Ethics Committee of Osaka Metropolitan University (Approval Number: #926). *Written informed consent was obtained from all patients*.

## Results

### Clinicopathological features

The clinicopathological characteristics of the 244 patients with HER2-negative breast cancer who underwent NAC and were included in this study are shown in Table 1. The median age was 55 years (range, 24–76 years), and all patients were female. The median tumor diameter was 30.3 mm (range, 9.2–119.8 mm), and skin infiltration was observed in 43 cases (17.6%). Axillary lymph node metastasis was found in 102 cases (59.3%), which was more than the number of cases without axillary lymph node metastasis. The ER-positive breast cancer was present in 113 cases (46.3%), while the PgR-positive breast cancer was present in 63 cases (25.8%). Higher Ki67 levels were present in 162 cases (66.4%).

**Table 1:** Clinicopathological features of 244 patients who were treated with preoperative chemotherapy

Parameters (n = 244)	Number of patients (%)
Age (years old)	55 (24–76)
Tumor size (mm)	30.3 (9.2–119.8)
Skin infiltration	
Negative/Positive	201 (82.4%)/43 (17.6%)
Lymph node metastasis	
Negative/Positive	70 (40.7%)/102 (59.3%)
Estrogen receptor positivity	
Negative/Positive	131 (53.7%)/113 (46.3%)
Progesterone receptor positivity	
Negative/Positive	181 (74.2%)/63 (25.8%)
HER2-status	
HER2-null*/Not HER2-null*	144 (59.0%)/100 (41.0%)
HER2-null*/HER2-ultralow**/HER2-low***	56 (23.0%)/88 (36.1%)/65 (26.6%)/35 (14.3%)
Ki67 index	
Low/High	82 (33.6%)/162 (66.4%)
Pathological response	
Non-pCR/pCR	176 (72.1%)/68 (27.9%)
Tumor- infiltrating lymphocytes density	
Low/High	147 (60.2%)/97 (39.8%)

*HER* Human epidermal growth factor receptor, *IHC* Immunohistochemistry, *ISH* In situ hybridization, *CR* Complete response

*HER2-null was defined as complete absence of staining

**HER2-ultralow was defined as ≤ 10% faint/weak incomplete membrane staining

***HER2-low was defined as IHC 1 + or IHC 2 + without ISH amplification

Regarding HER2 expression, 35 cases (14.3%) were IHC 2 + and FISH-, and 65 cases (26.6%) were IHC 1+, resulting in 100 cases (41.0%) that were HER2-low. Of the remaining 144 cases (59.0%) that were HER2-zero, 88 cases (36.1%) were classified as HER2-ultralow, and 56 cases (23.0%) were classified as HER2-null. Ninety-seven patients (39.8%) were classified into the higher TILs density group, and 68 patients (27.9%) achieved pCR.

### Correlation between HER2 expression and clinicopathological factors

In this study, HER2-negative cases not classified as HER2-null were defined as Not HER2-null. Among the 244 HER2-negative breast cancer patients, HER2-ultralow tumors showed significantly higher ER positivity compared with HER2-null tumors (51.8% vs. 19.6%, p < 0.001), and also tended to show higher PgR positivity (*p* = 0.048). In contrast, the HER2-null group was significantly younger (median 50.0 vs. 56.0 years, *p* = 0.004) and had significantly higher TIL density (50.0% vs. 36.8%, *p* = 0.016) compared with the Not HER2-null group **(**Table 2**)**.

**Table 2:** Comparison of clinicopathological features by HER2-status for HER2-negative breast cancer

Parameters	HER2 expression in HER2-negative breast cancer (n = 244)		*p* value	HER2-null* + HER2-ultralow*** (n = 144)		*p* value
	HER2-null* (n = 56)	HER2-low** + HER2-ultralow*** (n = 188)		HER2-null* (n = 56)	HER2-ultralow*** (n = 88)	
Age (years old)						
≤ 60	48 (85.7%)	123 (65.4%)	0.004	48 (85.7%)	60 (68.2%)	0.018
>60	8 (14.3%)	65 (34.6%)		8 (14.3%)	28 (31.8%)	
Tumor size (mm)						
≤ 20	6 (10.7%)	31 (16.5%)	0.290	6 (10.7%)	17 (19.3%)	0.169
>20	50 (89.3%)	157 (83.5%)		50 (89.3%)	71 (80.7%)	
Skin infiltration						
Negative	48 (85.7%)	153 (81.4%)	0.455	48 (85.7%)	73 (83.0%)	0.659
Positive	8 (14.3%)	35 (18.6%)		8 (14.3%)	15 (17.0%)	
Lymph node status						
Negative	13 (23.2%)	57 (30.3%)	0.302	13 (23.2%)	23 (26.1%)	0.693
Positive	43 (76.8%)	131 (69.7%)		43 (76.8%)	65 (73.9%)	
Estrogen receptor positivity						
Negative	42 (75.0%)	89 (47.3%)	< 0.001	42 (75.0%)	42 (47.7%)	0.001
Positive	14 (25.0%)	99 (52.7%)		14 (25.0%)	46 (52.3%)	
Progesterone receptor positivity						
Negative	47 (83.9%)	134 (71.3%)	0.058	47 (83.9%)	61 (69.3%)	0.048
Positive	9 (16.1%)	54 (28.7%)		9 (16.1%)	27 (30.7%)	
Ki67 index						
Low	16 (28.6%)	66 (35.1%)	0.363	16 (28.6%)	34 (38.6%)	0.216
High	40 (71.4%)	122 (64.9%)		40 (71.4%)	54 (61.4%)	
Pathological response						
Non-pCR	40 (71.4%)	136 (72.3%)	0.894	40 (71.4%)	64 (72.7%)	0.865
pCR	16 (28.6%)	52 (27.7%)		16 (28.6%)	24 (27.3%)	
Tumor- infiltrating lymphocytes density						
Low	26 (46.4%)	121 (64.4%)	0.016	26 (46.4%)	58 (65.9%)	0.021
High	30 (53.6%)	67 (35.6%)		30 (53.6%)	30 (34.1%)	

*HER *Human epidermal growth factor receptor, *CR* Complete response

*HER2-null was defined as complete absence of staining

**HER2-ultralow was defined as ≤ 10% faint/weak incomplete membrane staining

***HER2-low was defined as IHC 1 + or IHC 2 + without ISH amplification

*When focusing on the 144 HER2-zero tumors*, similar findings were observed: the HER2-null group was significantly younger (*p* = 0.018), had lower ER expression (*p* = 0.001), lower PgR expression (p = 0.048), and higher TIL density (p = 0.021) compared with the HER2-ultralow group. When stratified by hormone receptor status, HER2-null tumors in hormone receptor–negative cases (so-called triple-negative breast cancer, TNBC) were significantly younger than the Not HER2-null group (*p* < 0.001). No other significant correlations were observed in this stratified analysis》. (Supplementary Table 1).

### Prognostic impact of HER2 expression

**Fig. 1:**
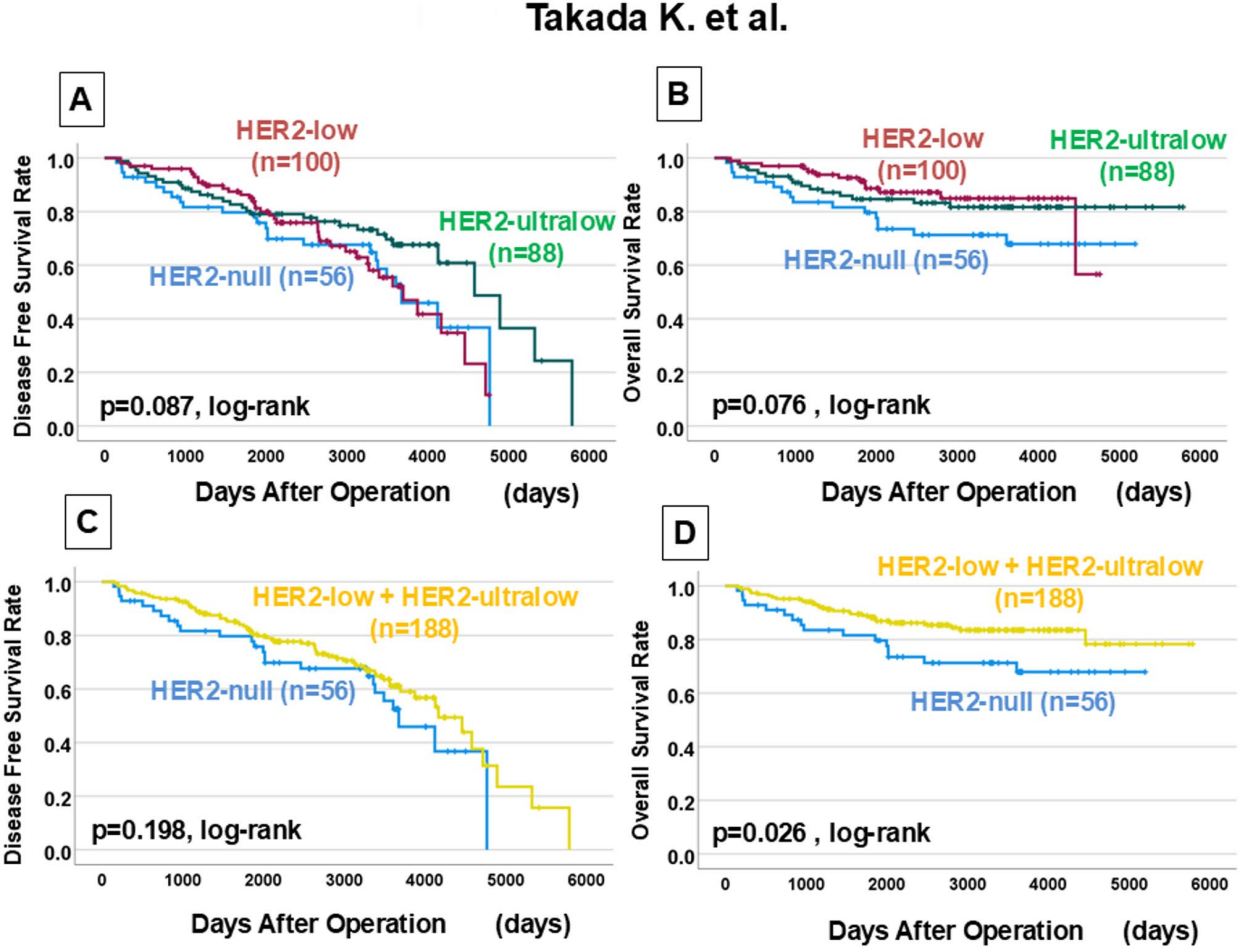
**A**) Disease-free survival (DFS) curves for *HER2-low (IHC 1 + or 2+/ISH–)*,* HER2-ultralow (≤ 10% faint/weak incomplete membrane staining)*,* and HER2-null (complete absence of staining)* groups (*p* = 0.087, log-rank test). **B** Overall survival (OS) curves for the three groups (*p* = 0.076, log-rank test). **C** DFS curves comparing HER2-null and Not HER2-null groups (*p* = 0.198, log-rank test). **D** OS curves comparing HER2-null and Not HER2-null groups, showing significantly worse survival for HER2-null cases (*p* = 0.026, log-rank test)

**Fig. 2:**
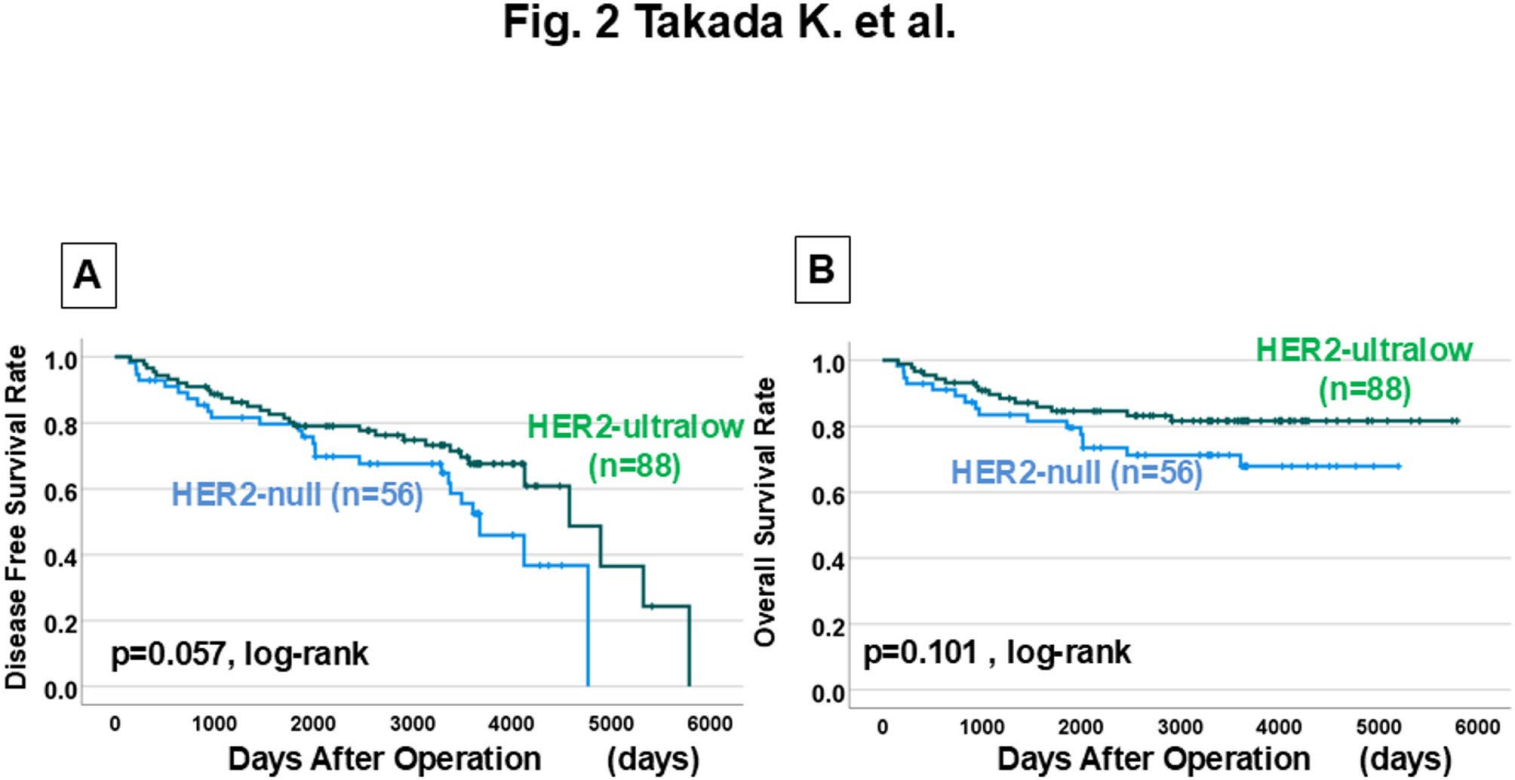
(**A**) DFS curves for the HER2-ultralow (n = 88) and HER2-null (n = 56) groups (p = 0.057, log-rank test). **B** OS curves for the same groups, with HER2-ultralow showing intermediate outcomes and HER2-null tending toward worse survival (p = 0.101, log-rank test) All figures are original and created by the authors

Log-rank tests were performed on 244 HER2-negative breast cancer patients, who were divided into three groups based on HER2 expression. No significant differences were found in DFS or OS (DFS: p = 0.087, log-rank OS: p = 0.076, log-rank) (Fig. 1A, B). However, >HER2-null tumors had significantly worse OS compared with Not HER2-null tumors (p = 0.026, log-rank) although DFS was not significantly different (p= 0.198, log-rank) (Fig. 1C, D). Among the 144 HER2-zero tumors, HER2-ultralow cases demonstrated an intermediate prognosis between HER2-low and HER2-null, with OS tending to be worse in HER2-null compared to HER2-ultralow (DFS: p = 0.057; OS: p = 0.101, log-rank) (Fig. 2A, B).

Univariate analysis of DFS in 244 patients with HER2-negative breast cancer showed no significant difference according to HER2 expression (*p* = 0.200, HR = 0.739) **(**Table 3**)**. On the other hand, univariate analysis of OS showed significant differences in skin infiltration (*p* < 0.001, HR = 2.971) and Not HER2-null (*p* = 0.029, HR = 0.505), and both were independent factors in multivariate analysis (skin infiltration: *p* = 0.002, HR = 2.797. Not HER2-null: *p* = 0.013, HR = 0.454). In the 144 HER2-zero breast cancer patients, no significant differences in DFS or OS were found in univariate analysis due to differences in HER2 expression (DFS: *p* = 0.059, HR = 0.591. OS: *p* = 0.106, HR = 0.559) (Supplementary Table 2). Similarly, in the 117 hormone receptor-positive breast cancer patients, no significant differences were observed in univariate analysis for DFS or OS (DFS: *p* = 0.879, HR = 1.075. OS: *p* = 0.237, HR = 0.511) (Supplementary Table 3), and the same was true for TNBC (DFS: *p* = 0.175, HR = 0.669. OS: *p* = 0.154, HR = 0.570) (Supplementary Table 4).

**Table 3:** Univariate and multivariate analysis with respect to disease-free survival and overall survival for HER2-negative breast cancer

Parameters	Disease-free survival						Overall survival					
	Univarite analysis			Multivariate analysis			Univarite analysis			Multivariate analysis		
	HR	95% CI	*p* value	HR	95% CI	*p* value	HR	95% CI	*p* value	HR	95% CI	*p* value
Age at opetation (yr)												
≤ 60 vs. > 60	1.259	0.806–1.968	0.311				1.001	0.524–1.914	0.997			
Tumor size (mm)												
≤ 20.0 vs. > 20.0	1.702	0.881–3.288	0.113				1.198	0.506–2.835	0.681			
Skin infiltration												
Negative vs. Positive	1.954	1.183–3.227	0.009	1.865	1.125–3.092	0.016	2.971	1.587–5.563	< 0.001	2.797	1.469–5.323	0.002
Lymph node status												
Negative vs. Positive	1.393	9.826–2.350	0.214				2.172	0.966–4.883	0.061	1.741	0.769–3.942	0.184
Estrogen receptor positivity												
Negative vs. Positive	0.796	0.521–1.215	0.290				0.619	0.335–1.143	0.125			
Progesterone receptor positivity												
Negative vs. Positive	0.502	0.292–0.864	0.013	0.397	0.231–0.682	< 0.001	0.554	0.257–1.193	0.131			
HER2-status												
Null* vs. HER2-low** + HER2-ultralow***	0.739	0.465–1.174	0.200				0.505	0.273–0.933	0.029	0.454	0.242–0.848	0.013
Ki67 index												
Low vs. High	1.425	0.910–2.231	0.122				1.676	0.847–3.320	0.138			
Pathological response												
Non-pCR vs. pCR	0.418	0.236–0.739	0.003	0.366	0.205–0.655	< 0.001	0.447	0.199–1.003	0.051	0.508	0.224–1.151	0.105
Tumor- infiltrating lymphocytes density												
Low vs. High	0.800	0.513–1.247	0.324				0.662	0.351–1.250	0.204			

*HER* Human epidermal growth factor receptor, *HR *Hazard ratio, *CI* Confidence intervals, *pCR* Pathological complete response

*HER2-null was defined as complete absence of staining

**HER2-ultralow was defined as ≤ 10% faint/weak incomplete membrane staining

***HER2-low was defined as IHC 1 + or IHC 2 + without ISH amplification

## Discussion

When examining the results of this study, the first thing to consider is that this study exclusively included cases treated with NAC. While this has the advantage of standardizing the therapeutic effect, it also introduces a bias in that NAC should be administered. Hormone receptor-positive breast cancer were relatively advanced cases of early breast cancer that require chemotherapy, whereas hormone receptor-negative breast cancer was excluded if the cancer is so small that NAC is not required, as well as elderly patients or patients with comorbidities who cannot tolerate NAC. As a result, the results of this study are different from the HER2 expression rate and clinicopathological factors reported in previous reports [6, 13, 14]. However, we consider this study to have the strength of examining prognosis in that it examined cases in which uniform NAC was administered.

Based on the results of the DESTINY-Breast04 Clinical Trials [5], there have been a number of studies examining the clinicopathological characteristics of HER2-low breast cancer. Many reports suggest that HER2-low breast cancer have higher ER expression and lower Ki67 expression compared to HER2-zero breast cancer [4, 20–32]. In addition, there are some reports that HER2-zero breast cancer patients were younger than HER2-low breast cancer patients [4, 21, 25, 26, 33–35]. Although there are few reports, a study has reported that TILs density increased with increased HER2 expression [19], while others have reported that there was no difference in TILs density depending on HER2 expression [4, 35]. It has been reported that HER2-zero breast cancer had higher TILs density than HER2-low breast cancer, regardless of ER expression [31], and only when limited to ER-negative breast cancer [36]. One study also reported that, although not significantly, HER2-zero breast cancer tends to have higher TILs density than HER2-low breast cancer [37], and there is variation regarding HER2 expression and TILs. Regarding prognosis, some reports have shown no difference between HER2-low and HER2-zero breast cancer [20, 21, 24, 25, 30, 36, 38–41], while others have shown that HER2-low breast cancer had better prognosis than HER2-zero breast cancer [22, 27, 29, 34, 42–47]. In particular, some reports have demonstrated potential molecular differences between HER2-zero and HER2-low breast cancer by analyzing PAM50-specific subtypes, highlighting the reasons for the differences in clinical prognosis [4, 42, 48]. Furthermore, gene expression analysis has confirmed that HER2-low breast cancers have a decreased expression of proliferation-related genes, tyrosine kinase receptor genes [4, 21], and androgen receptor [35]. Based on these results, two meta-analyses have reported that patients with hormone receptor-positive HER2-low breast cancer had a better prognosis than those with hormone receptor-positive HER2-zero breast cancer [23, 46]. Another meta-analysis reported that HER2-low breast cancer was associated with better DFS and OS than HER2-zero breast cancer, regardless of hormone receptor expression [33]. However, this study did not find a significant difference in prognosis between HER2-low breast cancer and HER2-zero breast cancer, which was thought to be due to the fact that this study was limited to cases that underwent NAC. In fact, in studies that were limited to cases that underwent NAC, there is a report that HER2-low breast cancer patients had a better prognosis [31], but most reports have not found a significant difference in prognosis [20, 21, 24, 39].

In contrast, evidence regarding HER2-ultralow remains limited. As with the HER2-low versus HER2-zero comparison, increasing HER2 expression has been associated with higher ER positivity [6, 13]. This study also demonstrated that HER2-ultralow tumors had significantly higher ER and PgR positivity compared with HER2-null, supporting the biological link between the HER2 and ER pathways [6, 49, 50]. Regarding age, our data showed that HER2-null patients were significantly younger than HER2-ultralow, consistent with prior reports that HER2-zero tumors occurred at a younger age than HER2-low tumors [4, 21, 25, 26, 33–35]. For TILs, however, results have varied. Chen et al. reported no difference in TIL density between HER2-ultralow and HER2-null [13], whereas our study found higher TIL density in HER2-null, particularly in TNBC. The discrepancy may be due to methodological differences (e.g., t-test vs. categorical cut-offs) and histological composition (Chen et al. included more non–invasive ductal carcinoma cases in the HER2-null group).

With respect to prognosis, Chen et al. did not observe significant survival differences by HER2 expression [13], whereas another study reported worse OS in HER2-null compared with HER2-ultralow [22]. Our findings are consistent with the latter, suggesting that HER2-null may represent a biologically distinct subgroup with adverse outcomes.

One of the limitations of this study is the use of HercepTest II (Dako) for HER2 IHC. Previous reports showed that staining results vary with antibody clones [51], and variability may be more pronounced in the ultralow range. Furthermore, even among experienced pathologists, interobserver reproducibility decreases as HER2 expression diminishes [4, 52]. Intratumoral heterogeneity of HER2 is another unresolved issue [6, 53, 54]. Since classification was based on pretreatment biopsy, discordance with the entire tumor burden cannot be excluded. Prognosis may also have been influenced by variations in adjuvant therapy, including postoperative chemotherapy.

Despite these limitations, our study provides novel evidence that HER2-ultralow tumors constitute a clinically relevant subgroup with distinct hormone receptor profiles and intermediate prognosis between HER2-low and HER2-null, in a uniform NAC-treated cohort.

## Conclusions

This study analyzed HER2-negative breast cancer with a refined classification. 

HER2-ultralow tumors represented a distinct subgroup, characterized by significantly higher hormone receptor positivity compared with HER2-null tumors.

 In contrast, HER2-null tumors were associated with younger age, lower hormone receptor expression, higher TIL density, and poorer survival. 

These findings highlight the clinical relevance of distinguishing HER2-ultralow within HER2-negative disease, particularly in the era of emerging ADC therapies. 

However, this study is limited by its retrospective single-institution design, sample size, and variability in HER2 assessment. 

Further large-scale, prospective studies are warranted to validate the clinical and prognostic significance of HER2-ultralow breast cancer.

## Supplementary Information

Supplementary Material 1.

## Data Availability

The datasets generated and analyzed during the current study are available in the Osaka Metropolitan University repository [https://ocu-omu.repo.nii.ac.jp/?page=1&size=20&sort=controlnumber] and can be accessed upon reasonable request.

## Abbreviations

BC: Breast Cancer
BCSS: Breast Cancer-Specific Survival
BMI: Body Mass Index
CI: Confidence Interval
CNB: Core Needle Biopsy
CT: Computed Tomography
DFS: Disease-Free Survival
ER: Estrogen Receptor
FISH: Fluorescence In Situ Hybridization
HER2: Human Epidermal Growth Factor Receptor 2
HR: Hormone Receptor
IHC: Immunohistochemistry
ISH: In Situ Hybridization
LVI: Lymphovascular Invasion
NG: Nuclear Grade
OS: Overall Survival
PgR: Progesterone Receptor
RFI: Recurrence-Free Interval
SLN: Sentinel Lymph Node
SLNB: Sentinel Lymph Node Biopsy
SLNM: Sentinel Lymph Node Metastasis
TILs: Tumor-Infiltrating Lymphocytes
TNBC: Triple-Negative Breast Cancer
US: Ultrasonography
VAB: Vacuum-Assisted Biopsy
