# Supplementary material for: Clinical characteristics and prognostic impact of HER2-ultralow breast cancer and tumor-infiltrating lymphocytes (TILs)
Source: BMC Cancer. 2025 Nov 28;25:1936. doi: 10.1186/s12885-025-15255-w (PMC12751392; doi:10.1186/s12885-025-15255-w)
Supplement: Supplementary file 1 — Supplementary Material 1. [file 12885_2025_15255_MOESM1_ESM.docx]

**Supplemental table 1. Comparison of clinicopathological features by HER2-status for intrinsic subtypes of breast cancer.**

| Parameters | HR+HER2-BC (*n* = 117) | | *p* value | TNBC (*n* = 127) | | *p* value |
| --- | --- | --- | --- | --- | --- | --- |
|  | HER2-null* (*n* = 15) | HER2-low** + HER2-ultralow*** (*n* = 102) |  | HER2-null* (*n* = 41) | HER2-low** + HER2-ultralow*** (*n* = 86) |  |
| Age (years old)  ≤ 60  > 60 | 11 (73.3%)  4 (26.7%) | 70 (68.6%)  32 (31.4%) | 0.712 | 37 (90.2%)  4 (9.8%) | 53 (61.6%)  33 (38.4%) | <0.001 |
| Tumor size (mm)  ≤ 20  > 20 | 0 (0.0%)  15 (100.0%) | 15 (14.7%)  87 (85.3%) | 0.112 | 6 (14.6%)  35 (85.4%) | 16 (18.6%)  70 (81.4%) | 0.580 |
| Skin infiltration  Negative  Positive | 12 (80.0%)  3 (20.0%) | 77 (75.5%)  25 (24.5%) | 0.702 | 36 (87.8%)  5 (12.2%) | 76 (88.4%)  10 (11.6%) | 0.926 |
| Lymph node status  Negative  Positive | 4 (26.7%)  11 (73.3%) | 26 (25.5%)  76 (74.5%) | 0.922 | 9 (33.3%)  18 (67.7%) | 31 (36.0%)  55 (64.0%) | 0.110 |
| Estrogen receptor positivity  Negative  Positive | 1 (6.7%)  14 (93.3%) | 3 (2.9%)  99 (97.1%) | 0.458 | -  - | -  - | - |
| Progesterone receptor positivity  Negative  Positive | 6 (40.0%)  9 (60.0%) | 48 (47.1%)  54 (52.9%) | 0.609 | -  - | -  - | - |
| Ki67 index  Low  High | 7 (46.7%)  8 (53.3%) | 45 (44.1%)  57 (55.9%) | 0.853 | 9 (22.0%)  32 (78.0%) | 21 (24.4%)  65 (75.6%) | 0.760 |
| Pathological response  Non-pCR  pCR | 13 (86.7%)  2 (13.3%) | 88 (86.3%)  14 (13.7%) | 0.967 | 27 (66.9%)  14 (34.1%) | 48 (55.8%)  38 (44.2%) | 0.282 |
| Tumor- infiltrating lymphocytes density  Low  High | 10 (66.7%)  5 (33.3%) | 78 (76.5%)  24 (23.5%) | 0.412 | 16 (39.0%)  25 (61.0%) | 43 (50.0%)  43 (50.0%) | 0.246 |

HER: human epidermal growth factor receptor. HR+HER2-BC: hormone receptor-positive and human epidermal growth factor receptor 2-negative breast cancer. TNBC: triple-negative breast cancer. HER: human epidermal growth factor receptor. CR: complete response.

*HER2-null was defined as complete absence of staining.

**HER2-ultralow was defined as ≤10% faint/weak incomplete membrane staining.

***HER2-low was defined as IHC 1+ or IHC 2+ without ISH amplification.

**Supplemental table 2. Univariate and multivariate analysis of HER2 status (HER2-null vs HER2-ultralow) for DFS and OS in HER2-negative breast cancer.**

| Parameters | Disease-free survival | | | | | | |  | Overall survival | | | | | | |
| --- | --- | --- | --- | --- | --- | --- | --- | --- | --- | --- | --- | --- | --- | --- | --- |
|  | Univarite analysis | | |  | Multivariate analysis | | |  | Univarite analysis | | |  | Multivariate analysis | | |
|  | HR | 95% CI | *p* value |  | HR | 95% CI | *p* value |  | HR | 95% CI | *p* value |  | HR | 95% CI | *p* value |
| Age at opetation (yr)  ≤ 60 vs > 60 | 1.241 | 0.668-2.304 | 0.494 |  |  |  |  |  | 1.056 | 0.472-2.361 | 0.895 |  |  |  |  |
| Tumor size (mm)  ≤ 20.0 vs > 20.0 | 1.394 | 0.629-3.091 | 0.413 |  |  |  |  |  | 1.271 | 0.445-3.634 | 0.654 |  |  |  |  |
| Skin infiltration  Negative vs Positive | 2.860 | 1.504-5.437 | 0.001 |  | 3.420 | 1.764-6.628 | <0.001 |  | 3.825 | 1.825-8.020 | <0.001 |  | 4.654 | 2.185-9.914 | <0.001 |
| Lymph node status  Negative vs Positive | 1.756 | 0.823-3.749 | 0.145 |  |  |  |  |  | 3.325 | 1.010-10.943 | 0.048 |  | 3.188 | 0.962-10.564 | 0.058 |
| Estrogen receptor positivity  Negative vs Positive | 0.598 | 0.335-1.068 | 0.082 |  | 1.053 | 0.536-2.067 | 0.881 |  | 0.612 | 0.288-1.300 | 0.202 |  |  |  |  |
| Progesterone receptor positivity  Negative vs Positive | 0.273 | 0.114-0.654 | 0.004 |  | 0.215 | 0.082-0.564 | 0.002 |  | 0.374 | 0.131-1.069 | 0.066 |  | 0.323 | 0.112-0.934 | 0.037 |
| HER2-status  Null vs HER2-ultralow | 0.591 | 0.342-1.021 | 0.059 |  | 0.610 | 0.339-1.098 | 0.099 |  | 0.559 | 0.277-1.132 | 0.106 |  |  |  |  |
| Ki67 index  Low vs High | 1.514 | 0.840-2.727 | 0.168 |  |  |  |  |  | 2.398 | 0.984-5.847 | 0.054 |  | 2.615 | 1.061-6.444 | 0.037 |
| Pathological response  Non-pCR vs pCR | 0.482 | 0.235-0.987 | 0.046 |  | 0.394 | 0.190-0.818 | 0.012 |  | 0.457 | 0.176-1.191 | 0.109 |  |  |  |  |
| Tumor- infiltrating lymphocytes density  Low vs High | 0.926 | 0.526-1.631 | 0.791 |  |  |  |  |  | 0.943 | 0.462-1.925 | 0.872 |  |  |  |  |

HER: human epidermal growth factor receptor. HR: hazard ratio. CI: confidence intervals. pCR: pathological complete response.

*HER2-null was defined as complete absence of staining.

**HER2-ultralow was defined as ≤10% faint/weak incomplete membrane staining.

***HER2-low was defined as IHC 1+ or IHC 2+ without ISH amplification.

**Supplemental table 3. Univariate and multivariate analysis of HER2 status for DFS and OS in hormone receptor-positive breast cancer.**

| Parameters | Disease-free survival | | | | | | |  | Overall survival | | | | | | |
| --- | --- | --- | --- | --- | --- | --- | --- | --- | --- | --- | --- | --- | --- | --- | --- |
|  | Univarite analysis | | |  | Multivariate analysis | | |  | Univarite analysis | | |  | Multivariate analysis | | |
|  | HR | 95% CI | *p* value |  | HR | 95% CI | *p* value |  | HR | 95% CI | *p* value |  | HR | 95% CI | *p* value |
| Age at opetation (yr)  ≤ 60 vs > 60 | 1.618 | 0.852-3.071 | 0.141 |  |  |  |  |  | 0.928 | 0.331-2.605 | 0.888 |  |  |  |  |
| Tumor size (mm)  ≤ 20.0 vs > 20.0 | 3.022 | 0.931-9.811 | 0.066 |  | 2.437 | 0.736-8.066 | 0.145 |  | 2.768 | 0.368-20.808 | 0.322 |  |  |  |  |
| Skin infiltration  Negative vs Positive | 1.543 | 0.752-3.168 | 0.237 |  |  |  |  |  | 1.807 | 0.677-4.819 | 0.237 |  |  |  |  |
| Lymph node status  Negative vs Positive | 1.701 | 0.709-4.078 | 0.234 |  |  |  |  |  | 3.033 | 0.697-13.195 | 0.139 |  | 2.953 | 0.678-12.858 | 0.149 |
| Estrogen receptor positivity  Negative vs Positive | 0.647 | 0.196-2.138 | 0.476 |  |  |  |  |  | 0.276 | 0.063-1.202 | 0.086 |  | 0.292 | 0.067-1.274 | 0.102 |
| Progesterone receptor positivity  Negative vs Positive | 0.433 | 0.231-0.814 | 0.009 |  | 0.489 | 0.258-0.926 | 0.028 |  | 0.596 | 0.235-1.510 | 0.275 |  |  |  |  |
| HER2-status  Null* vs HER2-low** + HER2-ultralow*** | 1.075 | 0.422-2.739 | 0.879 |  |  |  |  |  | 0.511 | 0.168-1.554 | 0.237 |  |  |  |  |
| Ki67 index  Low vs High | 1.222 | 0.670-2.226 | 0.513 |  |  |  |  |  | 1.673 | 0.628-4.460 | 0.304 |  |  |  |  |
| Pathological response  Non-pCR vs pCR | 0.582 | 0.207-1.635 | 0.304 |  |  |  |  |  | 1.3778 | 0.399-4.763 | 0.613 |  |  |  |  |
| Tumor- infiltrating lymphocytes density  Low vs High | 1.018 | 0.483-2.147 | 0.962 |  |  |  |  |  | 1.156 | 0.412-3.245 | 0.783 |  |  |  |  |

HR+HER2-BC: hormone receptor-positive and human epidermal growth factor receptor 2-negative breast cancer. HR: hazard ratio. CI: confidence intervals. HER: human epidermal growth factor receptor. pCR: pathological complete response.

*HER2-null was defined as complete absence of staining.

**HER2-ultralow was defined as ≤10% faint/weak incomplete membrane staining.

***HER2-low was defined as IHC 1+ or IHC 2+ without ISH amplification.

**Supplemental table 4. Univariate and multivariate analysis of HER2 status for DFS and OS in triple-negative breast cancer.**

| Parameters | Disease-free survival | | | | | | |  | Overall survival | | | | | | |
| --- | --- | --- | --- | --- | --- | --- | --- | --- | --- | --- | --- | --- | --- | --- | --- |
|  | Univarite analysis | | |  | Multivariate analysis | | |  | Univarite analysis | | |  | Multivariate analysis | | |
|  | HR | 95% CI | *p* value |  | HR | 95% CI | *p* value |  | HR | 95% CI | *p* value |  | HR | 95% CI | *p* value |
| Age at opetation (yr)  ≤ 60 vs > 60 | 0.954 | 0.508-1.792 | 0.885 |  |  |  |  |  | 1.040 | 0.452-2.393 | 0.926 |  |  |  |  |
| Tumor size (mm)  ≤ 20.0 vs > 20.0 | 1.107 | 0.494-2.476 | 0.805 |  |  |  |  |  | 0.812 | 0.305-2.166 | 0.678 |  |  |  |  |
| Skin infiltration  Negative vs Positive | 2.975 | 1.466-6.036 | 0.003 |  | 2.606 | 1.270-5.348 | 0.009 |  | 5.512 | 2.428-12.512 | <0.001 |  | 4.297 | 1.872-9.864 | <0.001 |
| Lymph node status  Negative vs Positive | 1.266 | 0.653-2.454 | 0.485 |  |  |  |  |  | 1.925 | 0.722-5.133 | 0.191 |  |  |  |  |
| HER2-status  Null* vs HER2-low** + HER2-ultralow*** | 0.669 | 0.375-1.195 | 0.175 |  |  |  |  |  | 0.570 | 0.263-1.235 | 0.154 |  |  |  |  |
| Ki67 index  Low vs High | 1.625 | 0.784-3.369 | 0.192 |  |  |  |  |  | 1.536 | 0.577-4.092 | 0.390 |  |  |  |  |
| Pathological response  Non-pCR vs pCR | 0.280 | 0.138-0.567 | <0.001 |  | 0.298 | 0.146-0.607 | <0.001 |  | 0.199 | 0.068-0.583 | 0.003 |  | 0.236 | 0.080-0.696 | 0.009 |
| Tumor- infiltrating lymphocytes density  Low vs High | 0.596 | 0.335-1.063 | 0.080 |  | 0.781 | 0.433-1.410 | 0.412 |  | 0.381 | 0.170-0.857 | 0.020 |  | 0.553 | 0.243-1.258 | 0.158 |

HR: hazard ratio. CI: confidence intervals. HER: human epidermal growth factor receptor. pCR: pathological complete response.

*HER2-null was defined as complete absence of staining.

**HER2-ultralow was defined as ≤10% faint/weak incomplete membrane staining.

***HER2-low was defined as IHC 1+ or IHC 2+ without ISH amplification.
